# Echinococcus Veterinorum1Read before the Missouri Valley Veterinary Association, February, 1899.

**Published:** 1899-04

**Authors:** S. Stewart

**Affiliations:** Kansas City, Mo.


### ECHINOCOCCUS VETERINORUM.[1]

By S. Stewart, V.S., M.D.,
KANSAS CITY, MO.

In the substance of this liver which is offered for your inspection, you will see a number of ovoid or spherical translucent bodies, varying in size from one inch to three inches in diameter. Some of them are apparently just beneath the capsule, while others are nearly hidden in the liver-structures. There are still others which are entirely within the substance of the organ. These bodies are cysts, and typical of the cystic phase of the echinococcus veterinorum, and constitute the hydatid disease of the older text-books. About five hogs in each one thousand slaughtered in this section of the country are bearers of the cystic echinococcus. These cysts may develop in any part or tissue, but are rarely found outside of the liver. The lungs are next in frequency of invasion. The dog is the host of the adult tapeworm, but nearly all animals may be the host of this cystic or larval form. In Iceland and continental Europe numerous cases of hydatid disease in mankind are recorded; a few cases have been reported in the United States. On account of this fact a thorough knowledge of this parasite is of sanitary importance to veterinarians.

Upon division of the walls of this cyst which I have selected you will note there escapes a quantity of clear, limpid fluid, and the wall is composed of two distinct layers. The outer layer is quite dense and intimately adherent to the surrounding structures; in fact, it appears to be a protecting wall developed from the tissues to resist the encroachment of the growth within. The inner layer is also quite thick and dense, and while everywhere closely coaptated to the outer wall, it is very feebly adherent to it, and is very readily separated and withdrawn through the incision just made. One distinctive characteristic of this membrane is a very persistent tendency of the cut edges to roll up. In fact, it is almost impossible to make it lie spread out upon a plane surface or on the palm of your hand. This powerful tendency to roll up is not possessed by any other membrane, according to some writers; hence when we find such a membrane, even when lacking particular characters soon

[1] Read before the Missouri Valley Veterinary Association, February, 1899.

to be mentioned, we may be reasonably certain the structure under observation is the mother-membrane of an echinococcus cyst.

A close inspection of the inner surface of this mother-membrane shows it to be studded over by a great number of very small grayish-white bodies or granules, which are loosely adherent and may be easily scraped away. I have mounted a number of these minute bodies on a microscopic slide, and, if you will examine them under a lens magnifying seventy-five to one hundred diameters, you will readily see that these little bodies (proligerous cysts) contain from fifteen to thirty pediculated tapeworm-heads. Each head is provided with two oval disks, called sucker-disks, and a rostellum of hooks. They constitute the fixation apparatus of the future worm. In nearly all the heads these organs are invaginated.

In these drawings pinned on the wall you have a magnified picture of atypical hydatid, and a representation of the more common modifications, such as the daughter and granddaughter cysts which develop from the mother-membrane, and may produce the little granular bodies containing the tapeworm-heads. Sometimes many small cysts are found, but in which the minute tapeworm-heads cannot be found. These are known as acephalo cysts.

This last drawing represents the adult worm as found in the intestines of the dog. The mature worm varies from one-sixth to one-fifth of an inch in length, and is composed of only three or four segments, the last one of which is nearly as long as the first three, is sexually mature and contains several thousand eggs. It is readily understood that a dog which eats two or three cysts such as we have just examined would become infested with a large number of these parasites. The heads attach themselves to the walls of the small intestines and become mature within sixty days, thereafter releasing the last or fourth segment as soon as ripe (ovulation completed), and continuously developing others. In this manner myriads of eggs are produced and pass out with the fecal discharges. The segments become dried, powdered, and the fragments are blown about by the wind. In this way the eggs find lodgement on all kinds of food-stuff, and with it are conveyed into the stomachs of animals and even man. The digestive processes in the stomach liberate the embryos in the eggs, which may permeate the walls of the intestines, enter the blood-stream, and find lodgement in any organ or part of the body, there to develop into cysts like these. Sometimes in man the cysts develop to immense proportions, or undergo degenerative changes which compromise the health and finally the life of the host.

Dogs infested with large numbers of these worms (tænia echinococcus) may suffer reflex nervous irritation with cerebral disturbances, including a state of frenzy, which may be mistaken for rabies. A post-mortem examination of the intestines of infected dogs would not reveal the presence of these worms to the casual observer, owing to their minute dimensions, but they are easily found by the close observer, and appear as short, yellowish threads or filaments attached by one end to the mucous membrane.

The prevalence of hydatids in swine indicates that many dogs, particularly those belonging to butchers and farmers, are infested with the adult worm. If butchers and farmers would cook or burn all organs of swine containing the cysts, instead of giving them to their dogs, the tænia echinococcus and its hydatid would soon be annihilated, and this menace to the public health be removed.

